# Cancer-associated fibroblasts in hepatocellular carcinoma: origins, heterogeneity, and therapeutic implications

**DOI:** 10.3389/fimmu.2025.1620075

**Published:** 2025-07-18

**Authors:** Xin Shi, Weixiong Zhu, Jianpeng Zhang, Chuanlei Fan, Jing Zhang

**Affiliations:** ^1^ Department of Oncology, Taizhou Second People’s Hospital Affiliated to Yangzhou University, Taizhou, Jiangsu, China; ^2^ The Second Hospital of Lanzhou University, Lanzhou, Gansu, China; ^3^ Department of Urology, The First Affiliated Hospital of Guangzhou Medical University, Guangzhou, Guangdong, China; ^4^ Department of Gastrointestinal Surgery, Jiangxi Province Hospital of Integrated Chinese and Western Medicine, Nanchang, Jiangxi, China

**Keywords:** hepatocellular carcinoma (HCC), cancer-associated fibroblasts (CAFs), tumor microenvironment (TME), therapeutic resistance, immune evasion

## Abstract

Hepatocellular carcinoma (HCC) is a globally prevalent malignancy. This disease often progresses rapidly, resulting in many patients being diagnosed at a late stage, making early detection and intervention a major clinical challenge. Postoperative recurrence and metastasis rates remain significantly high, and no effective prevention strategies are currently available. Cancer-associated fibroblasts (CAFs) are essential components in the reorganization of the tumor microenvironment (TME), as they can modulate cancer cell proliferation, migration, invasion, and chemoresistance through diverse mechanisms or signaling pathways, including the release of cytokines, remodeling of the extracellular matrix, and the evasion of the immune response. This review offers a detailed overview of the cellular origins, subtype diversity, and functional differences among CAFs. In addition, it depicts the expression profiles of key markers in various CAF subtypes and clarifies essential signaling pathways and mechanisms of CAFs. Additionally, we discuss current and future therapeutic strategies targeting CAFs in the context of HCC. This review provides critical insights into future studies on novel therapeutic approaches for CAFs.

## 1 Introduction

Liver cancer is the third leading cause of cancer-related mortality worldwide. Hepatocellular carcinoma (HCC) is the most common subtype of liver cancer, accounting for over half of all cases (1–3). Emerging evidence indicates that sustained hepatic inflammation and fibrotic remodeling foster a chronic pathophysiological environment conducive to malignant transformation. Chronic hepatitis, hepatic fibrosis, and HCC represent sequentially developed processes that are mediated by intricate multicellular crosstalk. The dynamic activation and stromal infiltration of cancer-associated fibroblasts (CAFs) are pivotal drivers of fibrotic progression and significantly contribute to hepatocarcinogenesis. Therefore, a more comprehensive understanding of the CAFs involved in the tumor microenvironment (TME) of HCC is necessary to decode their functional plasticity, identify targetable crosstalk mechanisms, and ultimately develop CAF-targeted therapies. In HCC, quiescent hepatic stellate cells (HSCs) are activated and transformed into myofibroblast-like CAFs, becoming one of the primary sources of CAFs; CAFs contribute significantly to the structural alterations of the microenvironment of the liver and are an essential component of TME (4–6). In the past, most researchers mainly focused on the malignant cells and neglected the stromal components. Recent advances in molecular and biological technologies have revealed dynamic bidirectional interactions between cancer cells and CAFs. Emerging evidence demonstrates that CAFs not only respond to oncogenic signals but also actively drive tumor progression through paracrine factor secretion (e.g., TGF-β, IL-6) and extracellular matrix remodeling, and act as critical collaborators in hepatocarcinogenesis (4, 7, 8). Cooperation between the tumor cells and CAFs facilitates supportive niches that enhance tumor aggressiveness. Furthermore, CAFs can influence the behavior of malignant cells and regulate the immune pattern within the context of the TME (9). Specifically, diverse CAF subtypes may differentially affect the effectiveness of immunotherapy outcomes through their capacity to modulate various types of immune cells and cancer cells. As indicated in many studies, the high expression of fibroblast activation protein (FAP) in CAFs is associated with an increasing infiltration of Tregs and M2 macrophages and a decreasing infiltration of CD8^+^ T cells and NK cells in the TME (10–12). It is expected to promote the proliferation and migration of HCC cells, and indicates a poor prognosis of HCC patients (10–12). In addition, CAFs can secrete many soluble factors and extracellular vesicles that modulate the immune response and are significantly correlated with the effectiveness of immunotherapy (4). Therefore, it is also increasingly important to understand the multifaceted effects of CAFs in HCC. Despite the promising role of CAFs as therapeutic targets, the evidence specifically targeting CAFs in HCC remains limited. However, insights from other solid cancers suggest that targeting CAFs may provide potential benefits in treating HCC.

This review aims to thoroughly summarize the cellular origins of CAFs within the context of HCC, the classification of CAF subtypes, and their diverse biological functions. CAFs are increasingly acknowledged as potential therapeutic targets as ongoing research continues to evolve and accumulate. By revealing the intricate interactions between CAFs and TME, we intend to summarize these insights to provide a basis for designing novel therapeutic strategies targeting CAFs to improve the therapeutic benefits and clinical outcomes of HCC patients.

## 2 Origins and heterogeneity of CAFs

**Figure 1:**
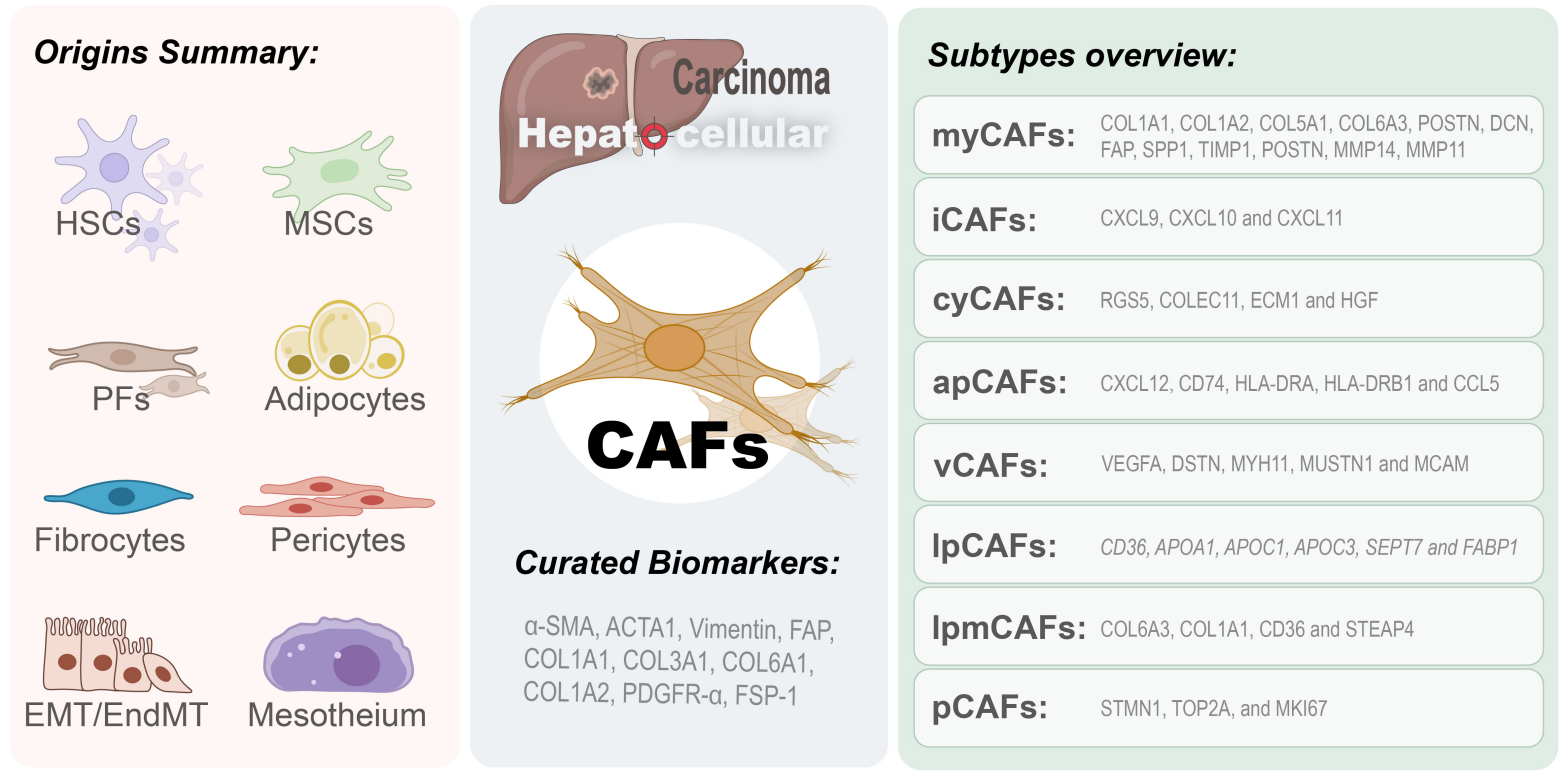
Origins and subtypes of CAFs in HCC. Cancer-associated fibroblasts in hepatocellular carcinoma microenvironment originate from multiple sources (left panel), including hepatic stellate cells (HSCs), mesenchymal stem cells (MSCs), portal fibroblasts (PFs), adipocytes, fibrocytes, pericytes, and cells derived from epithelial-to-mesenchymal transition (EMT) or endothelial-to-mesenchymal transition (EndMT). The right panel categorizes CAFs into eight subtypes: myofibroblast-like CAFs (myCAFs), inflammatory CAFs (iCAFs), cytokine-secreting CAFs (cyCAFs), antigen-presenting CAFs (apCAFs), vascular CAFs (vCAFs), lipid metabolism CAFs (lpCAF), lipid processing myofibroblast CAFs (lpmCAF), and proliferative CAFs (pCAFs).

CAFs in HCC derive from multiple cells, such as activated HSCs, resident fibroblasts, and mesenchymal stem cells (MSCs) sourced from bone marrow, as well as other stromal cells within the TME (e.g., endothelium, adipocytes) (4, 13, 14). This heterogeneity in origins contributes significantly to the intricacy and functional diversity of CAFs within the pathology of the disease (13) (refer to **Figure 1**). Throughout the hepatocarcinogenesis, chronic liver injury and advanced hepatic fibrosis function as critical driving forces. Over 90% of HCC instances emerge within the context of liver cirrhosis, and roughly one-third of patients suffering from cirrhosis ultimately advance to HCC (4). CAFs are recognized as the principal producers of collagen in the liver, demonstrating a strong correlation with the progression of HCC. HSC is a mesenchymal cell uniquely present in the liver and is the primary source of CAFs (13, 15, 16). HCC cells can secrete exosomal miRNA-21 into the environment, facilitating the transformation of HSCs into CAFs (17). Additionally, HCC cells can release sulfatase 2, enhance the transformation of HSCs into specific CAF types, and facilitate epithelial-mesenchymal transition (EMT) of cancer cells (18). Portal fibroblasts (PFs) are another essential origin of the CAFs in HCC. They are localized around the portal vein and keep the integrity of the biliary tree and portal vein intact (19). It was distinguished from HSCs by several expressed markers such as Thy1 and Fbln2. It drives myofibroblast generation throughout the initial phases of cholestatic liver fibrosis, whereas HSCs are the primary source of CAFs in later disease stages (19). Fibrocytes, originating from hematopoietic stem cells, demonstrate the presence of markers characteristic of both fibroblasts and hematopoietic cells, thus fulfilling an essential function in tissue regeneration and antigen presentation (14, 20). Following liver injury, these cells migrate from the bone marrow to the liver and spleen and differentiate into myofibroblasts under the regulation of CCR2, CCR1, TGF-β1, and LPS (4). EMT can result in aggressive CAFs with an altered cellular and molecular phenotype in HCC cells; expression of mesenchymal markers and fibroblast-specific proteins is increased with concomitant activation of specific pathways, such as TGF-β, and results in enhanced metastasis (21).

Other cell types with a potential CAF phenotype have also been recognized; these include hepatic sinusoidal endothelial cells (HSECs), MSCs, adipocytes, pericytes, and mesothelial cells (14, 21–26). The researchers concluded that HSEC can undergo endothelial-mesenchymal transition (EndMT), thus acquiring a fibroblast-like phenotype. MSCs can migrate to liver fibrosis and HCC microenvironments. A previous study found that co-culturing MSCs with HCC cells led to the acquisition of CAF properties and increased levels of tenascin-C and CXCL12 (4). A previous study showed that human adipose-derived mesenchymal stem cells acquired CAF-like characteristics after interaction with cancer cells when co-cultured with cancer cells (22), and the expression levels of a series of markers, such as α-SMA, porin, c-MYC, fibroblast growth factor receptor 1 (FGFR1), IL-6, IL-8, matrix metalloproteinase 2 (MMP2), vascular endothelial growth factor (VEGF), and tenascin-C, were significantly increased (22, 27–29).

In summary, the diverse origins of CAFs in HCC contribute to their complexity and functional roles in the TME, with activated HSCs and portal fibroblasts being primary sources. The interaction among these cells and diverse signaling pathways promotes HCC progression and increases tumor aggressiveness through multiple mechanisms.

## 3 Molecular subtypes and biomarkers of CAFs in HCC

Previous studies on HCC demonstrated that CAFs present in the tumor microenvironment show high heterogeneity, and different subtypes of CAFs display different biomarkers that exhibit distinct expression patterns. Although several biomarkers of CAFs have been reported, not all are CAF-specific because many are also present in other types of cells. It is interesting to explore the heterogeneity of CAFs and identify curated subcluster-specific markers to clarify how CAFs function in HCC. CAFs isolated from freshly resected HCC tumor tissues have shown MSC-like characteristics, as assessed by enhanced clonogenic potential and upregulation of CD73, CD90, CD105, CD44, CD13, CD29, and CD166 expression and downregulation of CD31, CD34, CD45, CD117, and HLA-DR expression. These cells retain multilineage differentiation capacity toward osteogenic, adipogenic, and pancreatic cell lineages (14). According to current research categorization conventions, CAFs can be classified into several common subtypes, including myofibroblastic CAF (myCAF), inflammatory CAF (iCAF), vascular CAF (vCAF), cytokines and growth factor-expressing CAF (cyCAF), antigen-presenting CAF (apCAF), proliferative CAF (pCAF), lipid processing myCAF (lpmCAF), and lipid metabolism and processing CAF (lpCAF) (16, 30, 31) (refer to **Figure 1**). Numerous analyses demonstrate that CAFs in HCC chiefly express an activated myofibroblastic phenotype. It was characterized by increased expression of specific markers, including α-smooth muscle actin (α-SMA), fibroblast activation protein (FAP), actin alpha cardiac muscle 1 (ACTA1), collagen type I alpha 1/2 chains (COL1A1, COL1A2), COL6A3, vimentin, fibroblast-specific protein-1 (FSP-1), platelet-derived growth factor receptors (PDGFRs), desmin, fibronectin, and collagen type I alpha chains (7, 14, 32–35). Other evidences show that the myCAF promote HCC progression via the COL-IDDR1 pathway, involving molecules such as COL1A1, COL1A2, COL5A1, COL6A3, POSTN, DCN, FAP, SPP1, MMP14, MMP11 and TIMP1 (16, 36, 37). The cyCAF displays an inhibitory role in the progression of HCC by modulating cytokines and growth factors, with significant molecular components comprising RGS5, COLEC11, ECM1, and HGF (16). The iCAFs are distinguished from other types by expressing classical signature genes including CXCL9, CXCL10, and CXCL11 (37). The vCAF is functionally linked to vascular smooth muscle contractile activity and calcium signaling pathways, involving VEGFA, DSTN, MYH11, MUSTN1, and MCAM (36–38). The apCAF assumes a function in antigen processing and presentation, possibly aiding in macrophage M2-like polarization and the recruitment of T lymphocytes, with pivotal mediators such as CD74, CXCL12, HLA-DRA, CCL5, and HLA-DRB1 (36–38). Proliferative CAFs exhibit high expression of STMN1, TOP2A, MKI67, and other markers involved in proliferation and the cell cycle (37). lpCAFs are involved in the remodeling of protein-lipid complexes, the metabolism of fatty acids (specific expression of CD36, APOA1, APOC1, APOC3, SEPT7, and FABP1), and the recruitment of CD33^+^ myeloid-derived suppressor cells (MDSCs). LpmCAFs are involved in processes such as extracellular matrix dynamics, cholesterol metabolism, and lipid metabolism that promote the growth of HCC, with purposely highly expressed markers including COL6A3, COL1A1, CD36, and STEAP4 (16, 36, 37).

A series of recent single-cell RNA-sequencing (scRNA-seq) studies have defined multiple subpopulations, molecular features, and functions of fibroblasts in HCC that differ from classical common subtypes. The investigation by Meng et al. demonstrated significant and distinct variations in fibroblast properties, attributed to the gene expressions of COL4A1, COL1A1, and COL6A2, all of which are pivotal in the elaborate biological framework of extracellular matrix-receptor crosstalk (39). Their investigation further disclosed that COL1A1 and ITGA2 exhibit the strongest interactions through communication between CAFs and malignant cells (35). CD36^+^ CAFs in hepatocellular carcinoma (HCC) exhibit upregulated lipid metabolism-driven immunosuppression by impairing cytotoxic T lymphocyte activity and elevating PD-1 expression, as demonstrated in murine models (36). Wang and colleagues categorized CAFs into a total of six distinct subtypes in HCC utilizing scRNA gene expression profiles: STMN1^+^ CAFs, CXCL12^+^ CAFs, MYH11^+^ CAFs, SEPT7^+^ CAFs, POSTN^+^ CAFs, and CD36^+^ CAFs, and they specifically focused on the newly discovered POSTN^+^ CAF subpopulation, which plays a crucial role in promoting the progression of HCC through the activation of the ECM, hypoxia and TGF-β signaling pathways (37). The study by Yan et al. reveals that YAP1-positive fibroblasts linked to oncogenesis stimulate the swift proliferation of HCC cells by enhancing the expression of genes associated with matrix rigidity (40). Liu and colleagues identified three CAF subtypes (HLA-DRB1, MMP11, and VEGFA), with VEGFA+ CAFs activated by the hypoxic microenvironment, linked to poorer prognosis, and promoting tumor angiogenesis through interactions with capillary endothelial cells (38).

In summary, the expression of CAF biomarkers is highly heterogeneous and largely depends on the specific CAF subtypes investigated in these studies. The heterogeneity of CAFs is not only manifested by the expression of these biomarkers but is also closely associated with their functional roles in the TME (4, 13, 14). Therefore, the specific biomarkers and potential functions need further investigation in CAF-targeted therapies. Only when the specificity and functional complexity of CAF biomarkers are fully revealed and linked can targeted therapies offer hope for treatment. Future research should integrate multi-omics analyses and functional validation to clarify subtype-specific mechanisms and therapeutic targets.

## 4 Biological roles of CAFs in HCC

**Figure 2:**
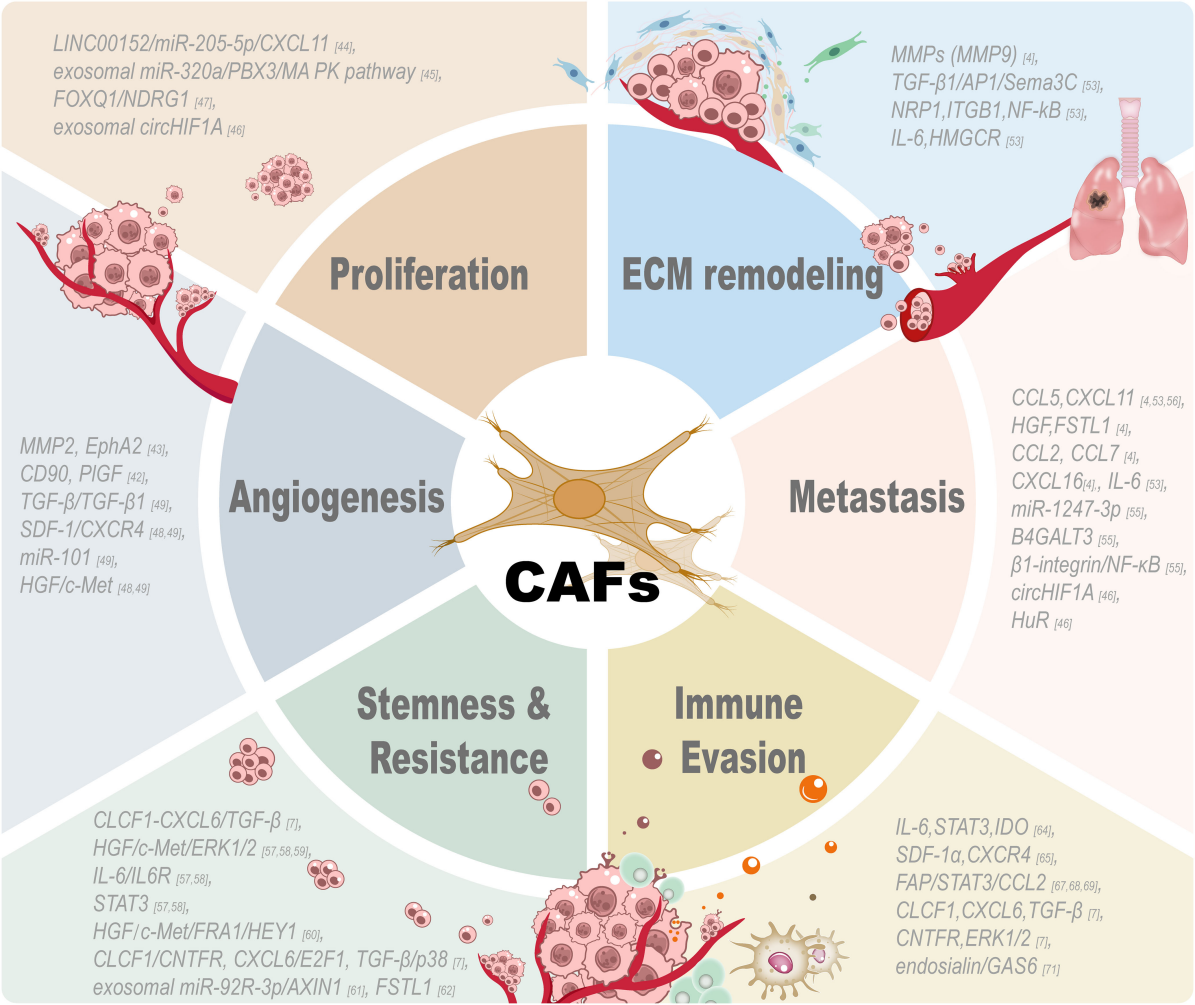
Tumor-associated functions of CAFs. CAF participates in multiple aspects of the tumor microenvironment and exerts great effects on these processes: tumor cell proliferation, extracellular matrix remodeling, angiogenesis, metastasis, stemness, drug resistance and immune evasion. CAF promotes the proliferation of tumor cells by secreting growth factors and signaling proteins. CAFs are indispensable for the stroma and have induced tumor invasion by remodeling the ECM and tumor architecture changes. Angiogenic factors produced by CAFs can promote the growth of new blood vessels and tumor vascularization by stimulating the formation of new blood vessels. CAFs promote the tumor cell metastasis by secreting chemokines to induce the migration of tumor cells. CAFs can maintain the cancer stem cell property and drug resistance of tumor cells through different signaling pathways. CAFs can modulate different kinds of immune cells and participate in the immune evasion process by regulating the immunosuppressive microenvironment created by CAFs to help tumor cells escape from the attack of immune cells. All these results reveal the diversity and important role of CAFs in tumor biology and suggest that targeting these pathways may be an effective approach to treat cancer.

CAFs can significantly impact cancer cells and neighboring cell populations, altering the TME and influencing the direction of disease evolution. Previous studies have elucidated that CAFs can promote cell proliferation, self-renewal capacity, migratory behavior, invasive potential, drug resistance, and metastatic spread through multiple signaling pathways (14) (refer to **Figure 2**).

### 4.1 CAFs facilitate cancer cell proliferation and vascular mimicry

Increasing evidence of research points out that CAFs are essential factors in liver cancer progression and boost the growth of malignant cells (7, 30, 41, 42). Liu et al. reported that the LINC00152/miR-205-5p/CXCL11 signaling mechanism in CAFs is pivotal in determining the traits of malignant cell proliferation, validating that LINC00152 increases CXCL11 production by binding miR-205-5p (43). Zhang et al. provided evidence that the reduction of exosomal miR-320a derived from CAFs stimulates the growth and metastasis of HCC through PBX3 targeting and MAPK signaling pathway inhibition (44). Hypoxic carcinoma-associated fibroblasts (H/CAFs) significantly enhance the replication of tumor cells, contributing to the increases in tumor size and development (45). The FOXQ1/NDRG1 axis promotes the initiation of HCC by modulating the crosstalk between CAFs and tumor cells, which forms a circular reinforcement loop to attract HSCs and encourage tumor proliferation (46). Vasculogenic mimicry (VM) is the ability of aggressive tumor cells to generate vascular-like structures by self-deformation and remodeling of the ECM. CAFs enhance VM formation in HCC by upregulating the expression of MMP2 and EphA2 through the secretion of factors; in addition, the increased vimentin and α-SMA expression in VM-positive tissues was significantly associated with the clinicopathological traits of HCC samples, including tumor diameter and Edmondson grade (42). Furthermore, CAFs activate the CD90/placental growth factor (PlGF) axis with high CD90 expression and PlGF secretion to promote neoangiogenesis (upregulation of CD34, CD31, and CD105) and are correlated with poor prognosis (41). Thus, targeting the CAFs-PlGF axis may inhibit pro-angiogenic TME. To regulate the arrangement of VM in the TME, CAFs release SDF-1, TGF-β, and HGF, which combine with their corresponding membrane receptors on tumor cells, including CXCR4, TGF-βR1, and c-Met, to modify the plasticity of HCC cells (47, 48). CAFs promote VM in HCC via TGF-β/SDF1-induced VE-cadherin/MMP2/LAMC2 axis, while miR-101 can suppress VM by dual targeting TGF-βR1/Smad2 in HCC cells and SDF1 in CAFs for anti-metastasis therapy (48). Understanding the participation of CAFs in alternative vascularization, including angiogenesis-dependent procedures, may improve the development of targeted therapies for HCC. Moreover, CAFs have been shown to induce metabolic reprogramming in HCC cells, which is crucial for tumor progression. CAFs can secrete various metabolites and growth factors, such as lactate and glutamine, to regulate the metabolic landscape of the TME. For example, CAFs-derived lactate can be taken up by cancer cells and used as a fuel source, promoting their proliferation and survival. CAFs also secrete HGF, which activates the c-Met receptor in HCC cells, which leads to the activation of downstream signaling pathways that drive metabolic reprogramming. Targeting these metabolic interplays may offer novel therapeutic opportunities for HCC treatment.

### 4.2 CAFs promote extracellular matrix remodeling

CAFs have been reported to promote malignant progression in various cancers through modulation of the tumor microenvironment by altering the ECM, which is composed of a lot of proteins maintaining tissue architecture and cell growth; alterations in the ECM promote tumor initiation, invasion, metastasis, and drug resistance in solid tumors (4). Notably, Schrader et al. reported that a stiffer augmented matrix of ECM facilitates cellular proliferation and resistance to chemotherapeutic drugs in HCC cells. In contrast, a more yielding ECM setting enables reversible dormancy and the acquisition of stem cell-like phenotypes, further demonstrating the pivotal role of the mechanical properties of ECM in tumor progression and patient outcomes (49). Matrix metalloproteinases (MMPs), such as MMP9, are essential components of the mechanism of extracellular matrix degradation, and many researchers have demonstrated that these enzymes play a key role in the initial stage of EMT during fibrosis (4). Recent reports have indicated that mechanical forces generated by premetastatic cancer cells and the contractility of CAFs are involved in the remodeling of the ECM and intercellular interactions, which influence the growth and progression of HCC and make these two components potential targets for tumor therapy (50, 51). The results from the study by Peng et al. demonstrated that, in HCC, CAFs of different types can release TGF-β1, which can activate AP1 signaling pathways and upregulate the expression of Sema3C (52). This leads to the contraction of the ECM and the activation of HSCs (52). Mechanistically, Sema3C colludes with NRP1 and ITGB1 on HSCs, triggering the downstream NF-kB signaling cascade, ultimately leading to the output of IL-6 and the upregulation of HMGCR gene.

### 4.3 CAFs promote metastasis of HCC

CAFs promote pulmonary metastasis of HCC via paracrine CCL5, CXCL11, HGF, and FSTL1 (4, 52). In preclinical mouse models, CCL5 released from CAFs could inhibit the degradation of HIF1α and increase the expression of ZEB1. Biological activity shown above greatly promotes EMT, a basic cellular process that plays a vital role in the development and progression of many cancers and promotes lung metastasis from HCC (53). CAF-Factors, including CXCL16, IL-6, CCL2, CCL7, heparin-binding EGF-like growth factor, and POSTN, promote the invasion of HCC in co-culture models by stimulating the secretion of chemokines that activate the hedgehog and TGF-β pathways for their pro-metastatic effects, which remain to be verified *in vivo* (4, 52). Fang et al. found that exosomal miR-1247-3p, secreted by tumor cells, could promote the activation of CAFs by inducing its activation, leading to lung metastasis of HCC by targeting B4GALT3 and enhancing β1-integrin-NF-κB signaling pathway (54). Liu et al. found that CAF-derived CXCL11 enhanced HCC cells migration and metastasis by the circUBAP2/miR-4756/IFIT1/3 signaling pathway; CAFs participate in cancer development by promoting the invasion and metastasis of HCC cells (55). Shang et al. have revealed that exosomal circHIF1A, derived from hypoxic cancer-associated fibroblasts (H/CAFs), can upregulate PD-L1 expression in HCC cells. This action promotes HCC progression and immune evasion, primarily by inhibiting the cytotoxic activity of CD8^+^ T cells, and the process is conditionally dependent on Hu antigen R (HuR) (45).

### 4.4 CAFs induce cancer stemness and mediate drug resistance in HCC

CLCF1-CXCL6/TGF-β axis mediates the interaction between tumor cells, CAFs, and tumor-associated neutrophils to promote HCC development and cancer stemness (7). CAFs also promote the stemness of CD24^+^ liver cancer cells by secreting HGF and IL-6, which can activate STAT3 phosphorylation at the Tyr705 site, to drive self-renewal, chemotherapy resistance, and distant metastasis (56). CAFs promote stem cell-like properties in malignant cells by activating the IL-6/STAT3/Notch axis, driving tumor aggressiveness and poor prognosis (57). In a study conducted by Lai et al., they found that the IL-6/STAT3 signaling pathway regulates the DNMT3b enzyme, and it can facilitate the development of resistance to the chemotherapeutic agent sorafenib by promoting the expression of OCT4, which, at the same time, led to a poor prognosis and outcome in HCC (57). Hepatocyte growth factor (HGF) from CAFs drives HCC stemness and chemoresistance through FRA1-dependent signaling, enhancing spheroid formation, upregulating stem cell markers (CD44/EpCAM), and conferring cisplatin and sorafenib resistance, as evidenced by multiple preclinical studies (56, 58, 59). Additional research has indicated that CAFs in HCC enhance tumor-initiating cell characteristics and therapeutic resistance by releasing HGF and IL-6, which activate the FRA1 and Notch signaling pathways, respectively (56, 60, 61). Upon further investigation, it was found that CLCF1 acts as a cytokine that promotes cancer cell stemness in HCC. Its biological functions are associated with ciliary neurotrophic factor receptor (CNTFR) and downstream CXCL6/E2F and TGF-β/MAPK signaling pathways, which promote the expression of stemness-associated genes and tumorigenesis (7). As reported by Su et al., exosomal miR-92a-3p secreted by CAFs fosters the proliferation of tumors and stemness in HCC via the Wnt/β-catenin signaling cascade by downregulating AXIN1 expression (62). Loh et al. found that follistatin-like 1 (FSTL1) secreted by activated fibroblasts drives stem cell characteristics, metastasis, and sorafenib resistance in HCC through the TLR4/AKT/mTOR/4EBP1 signaling axis. High FSTL1 level in FAP positive fibroblasts is associated with poor patient prognosis, and targeting FSTL1 can inhibit tumor malignancy and extend survival in tumor-bearing mice (60). Using spatial multi-omics technology, a novel subpopulation of cancer-associated fibroblasts (F5-CAF, marked by COL1A2/COL4A1/CTGF/FSTL1) was identified in HCC, which is spatially located around tumor nests and co-localizes with cancer cells exhibiting high stemness, driving liver cancer progression and poor prognosis by facilitating the existence of cancer stem cells (61).

### 4.5 CAFs facilitate immune evasion in TME

CAFs recruit immune cells, including monocytes, neutrophils, and dendritic cells, and aid these cells to adopt an immunosuppressive phenotype, thereby facilitating immune evasion in HCC (63–65). Research shows that HCC-derived CAFs facilitate the generation of regulatory DCs via IL-6-mediated STAT3 activation, leading to increased IDO production and T-cell regulation (63). CAFs attract monocytes and induce their transformation into MDSCs via the SDF-1α/CXCR4 pathway and IL-6-mediated STAT3 activation, impairing T-cell proliferation and promoting HCC progression (64). Cheng et al. further explained that CAFs linked with HCC induce programmed death-ligand 1-positive (PD-L1^+^) neutrophils via the interleukin-6-signal transducer and activator of transcription 3 (IL-6/STAT3) signaling pathway, thereby augmenting immune evasion by hindering T-cell functionality (65). Research by Yang et al. provided *in vivo* insights that validate the role of CAFs in promoting HCC progression through the recruitment of MDSCs via the fibroblast activation protein (FAP)-orchestrated urokinase-type plasminogen activator/receptor (uPAR)/focal adhesion kinase (FAK)/Src/Janus kinase 2 (JAK2) axis, which then activates STAT3 and triggers the expression of C-C motif chemokine ligand 2 (CCL2), thereby highlighting the vital importance of CAFs in the development of an immunosuppressive TME (66, 67). Recent studies have identified some factors released by CAFs in liver cancer, like IL-6, CXCL12, IL-8, and CCL-2, which inhibit CD8^+^ T cells and facilitate M2 macrophage differentiation (68, 69). H/CAFs induce immune suppression by inhibiting the cytotoxicity and activity of CD8^+^ T cells, allowing HCC cells to evade immune surveillance and persist in the TME (45). In the study by Song et al., they found that cytokines from CAFs, like CLCF1, can enhance the interaction between HCC cells, CAFs, and cancer-associated neutrophils and increase N2 neutrophil infiltration and tumor proliferation by combination with CNTFR receptors (7). At the same time, they found that ERK1/2 is the core regulator in the positive feedback loop, which strengthens the signaling pathways of CLCF1, CXCL6, and TGF-β, and effectively promotes the malignant transformation of HCC (7). Furthermore, Yang et al. demonstrated that CAFs induce immunosuppression via promoting macrophage polarization switching to M2 type through endosialin/CD68-mediated release of GAS6 factor, and the anti-endosialin antibody IgG78 blocked this tandem via inducing lysosomal degradation of glycosylated endosialin, which could significantly inhibit CAF-induced M2 infiltration and tumorigenesis in preclinical models (70). In conclusion, CAFs induce immunosuppression through direct or indirect contact with immune cells, successfully modulating immune cells into a tolerant phenotype, and then promoting immune evasion.

## 5 Therapeutic strategies targeting CAFs in HCC

Currently, there are no FDA-approved drugs specifically targeting CAFs in HCC. Some FDA-approved systemic therapies for HCC, such as anti-angiogenic agents (e.g., sorafenib, lenvatinib, cabozantinib) and immune checkpoint inhibitors (e.g., nivolumab, pembrolizumab), may indirectly affect CAF activity, but they are not specifically designed to target CAFs (13). CAF-targeted therapies can inhibit tumor progression. These therapeutic strategies prevent CAF formation and infiltration by eliminating CAFs, normalizing their functions, and inducing a tumor-suppressive phenotype. Currently, most studies focus on inhibiting the paracrine effects of CAFs. However, few druggable CAF-related targets have been identified, and most of these studies are still in preclinical stages (13, 14). For example, Liu et al. reported that combining IL-6 blockade and anti-PD-L1 therapy could improve anti-tumor immunity and overcome resistance to immunotherapy in the mouse HCC model. This study proposes a novel therapeutic strategy to enhance PD-L1 inhibitor efficacy in HCC (71). A preclinical study demonstrates that the TGF-β receptor inhibitor LY2109761 can downregulate the connective tissue growth factor (CTGF), disrupt tumor-stroma interactions, and significantly inhibit malignant progression, which suggests potential clinical benefits for targeting CTGF (72). Furthermore, some early clinical studies are also ongoing. A randomized phase I study comparing the multi-targeted inhibitor dovitinib (VEGFR, PDGFR, FGFR) versus sorafenib (VEGFR, PDGFR) in advanced HCC demonstrated comparable median overall survival and time to progression, with subgroup analysis suggesting improved survival for dovitinib-treated patients with baseline sVEGFR1 or HGF below median levels; however, no superiority over sorafenib was observed, and no phase 3 trial is planned (73). A Phase I study of H3B-6527 (NCT02834780), a selective FGFR4 inhibitor, demonstrated a favorable safety profile and encouraged clinical activity in patients with advanced HCC who had previously undergone multiple therapies. With a recommended Phase II dose of 1000 mg daily, the study reported an overall response rate of 16.7% and a clinical benefit rate of 45.8% among heavily pretreated HCC patients (74). A Phase 2 trial assessed the efficacy of galunisertib (NCT01246986), a TGF-β1 antagonist, with sorafenib in individuals with late-stage HCC. The results indicated acceptable safety and efficacy, with responders exhibiting significantly longer overall survival than non-responders (75). A recent trial (CTR20230372) demonstrated that combining the oral STING agonist MSA-2 with the anti-TGF-β/PD-L1 bispecific antibody YM101 could enhance anti-tumor immunity in immune-silent tumor models (e.g., immune-excluded and immune-desert) (76). This combinational strategy improves dendritic cell maturation, T cell activation, and tumor-infiltrating lymphocyte activity, which could significantly inhibit malignant progression. A Phase Ib/II trial focused on the safety and therapeutic efficacy of BLU-554 (NCT04194801), an antagonist of FGFR4. This study was performed alongside CS1001, a monoclonal PD-L1 antibody, specifically for individuals with locally progressed or metastatic HCC (77). Although most CAF-targeted therapies are still in preclinical stages, some existing data have demonstrated their therapeutic potential in HCC. Further studies are required to validate efficacy and optimize combination strategies for comprehensive HCC treatment.

## 6 Different techniques to identify CAFs in HCC

ScRNA-sequencing technique reveals the heterogeneity of CAFs and their distinct gene expression patterns, and imitates the developmental trajectories of CAFs, and depicts their interactions with other cells. However, the limitations of scRNA-seq analysis are mainly attribute to high technical requirements and costs, complex experimental procedures and data analysis. In addition, the lineage tracing technique uses specific genetic tools or markers to track the origin and differentiation processes of CAFs. This method provides insights into the origins of CAFs in HCC and helps clarify the mechanisms of CAF activation and differentiation. However, the complex technical implementation and difficulty in selecting appropriate lineage-specific markers restrict its widespread utility. Immunohistochemistry (IHC) is also widely used to detect specific cell types in tumors. This technique can use specific antibodies to detect CAF-related proteins in HCC tissue sections and visualize their localization, distribution, and relative content. IHC allows direct observation of CAF distribution and quantity in HCC tissues, and it can be combined with histopathological analyses. However, IHC staining results depend on antibody specificity and quality, and can be affected by subjective interpretation. Overall, each method for identifying and characterizing CAFs in HCC has its strengths and limitations. In practice, these methods are often combined to complement one another, and these combinations enable a more accurate detection and comprehensive understanding of CAFs in HCC.

## 7 Conclusion and future direction

CAFs are critical in modulating HCC development and progression by dynamically regulating TME through interactions with cancer cells, immune cells, and ECM (4, 13, 14). Due to their high functional heterogeneity caused by their diverse cellular origins, CAFs have been reported to exert pleiotropic effects in cancer development, including tumor promotion, metastasis, immune evasion, and therapy escape. Recently, more CAF subpopulations with various tumor-modulating functions were discovered by applying advanced scRNA-seq and spatial transcriptomics technologies, providing insight into the biomarker diversity and molecular crosstalk among CAFs (35, 38). The therapeutic targeting of CAF subpopulations or their secreted molecules, such as cytokines, exosomes, and downstream signaling pathways, especially in combination with immune checkpoint inhibitors, has been demonstrated in preclinical studies (71, 76). However, these approaches still face many challenges in clinical translation because of our limited knowledge of CAF biology in HCC, high heterogeneity, and specific functional contexts. We believe that future research should focus on the following questions to solve the remaining mysteries of CAF subsets for precise therapies, including the unresolved questions on epigenetic regulation of CAF transition, spatial distribution pattern of CAFs in TME, and specific biomarker validation for distinguishing tumor-promoting from tumor-restraining subtypes. Integrating multi-omics approaches with advanced preclinical models will bridge gaps between *in vitro* findings and clinical relevance. Single-cell and spatial transcriptomics are critical for uncovering the architectural context and clarifying rare CAF subsets. Solving the technical issues involved in CAF extraction, the specific activity of antibodies, and the validation of results *in vivo* will be necessary for translating preclinical findings into safe and efficacious clinical applications. Moreover, standardizing the nomenclature associated with CAF subtypes and instituting public transparency regarding defining markers will enhance the connectivity and comparability among research teams and studies, thereby expediting the identification of novel biomarkers and the investigation of prospective mechanisms. From a clinical practice perspective, therapeutic approaches targeting CAFs must rely on the specific elimination of certain subpopulations or the intelligent reprogramming of the TME to distinguish different “demon” CAF subtypes while simultaneously preserving normal fibroblast and other stromal function with limited functional impairment. Using multiple approaches, either inhibiting the dialogue between CAFs and immune cells, altering the ECM remodeling pathway, or promoting differentiation of precursor cells, would be expected to enhance the efficacy of agents and reduce the resistance. Assessing and classifying individuals based on CAF characteristics and the TME may increase the success rate of immunotherapy for HCC.

## Glossary

apCAF: antigen-presenting CAF
CAF: cancer-associated fibroblasts
cyCAF: cytokines and growth factor-expressing CAF
DCs: dendritic cells
ECM: extracellular matrix
EMT: epithelial-mesenchymal transition
EndMT: endothelial-mesenchymal transition
HCC: hepatocellular carcinoma
H/CAFs: hypoxic carcinoma-associated fibroblasts
HSCs: hepatic stellate cells
iCAF: inflammatory CAF
lpCAF: lipid metabolism and processing CAF
lpmCAF: lipid processing myCAF
MDSCs: myeloid-derived suppressor cells
MMP: matrix metalloproteinase
MSCs: mesenchymal stem cells
myCAF: myofibroblastic CAF
pCAF: proliferative CAF
PDGFRs: platelet-derived growth factor receptors
PFs: portal fibroblasts
PlGF: placental growth factor
TME: tumor microenvironment
vCAF: vascular CAF
VM: vasculogenic mimicry

## References

[1] Sung H Ferlay J Siegel RL Laversanne M Soerjomataram I Jemal A. Global cancer statistics 2020: GLOBOCAN estimates of incidence and mortality worldwide for 36 cancers in 185 countries. CA Cancer J Clin. (2021) 71:209–49. doi: 10.3322/caac.21660, PMID: 33538338

[2] Rumgay H Ferlay J de Martel C Georges D Ibrahim AS Zheng R. Global, regional and national burden of primary liver cancer by subtype. Eur J Cancer. (2022) 161:108–18. doi: 10.1016/j.ejca.2021.11.023, PMID: 34942552

[3] Bray F Laversanne M Sung H Ferlay J Siegel RL Soerjomataram I. Global cancer statistics 2022: GLOBOCAN estimates of incidence and mortality worldwide for 36 cancers in 185 countries. CA Cancer J Clin. (2024) 74:229–63. doi: 10.3322/caac.21834, PMID: 38572751

[4] Ying F Chan MSM Lee TKW. Cancer-associated fibroblasts in hepatocellular carcinoma and cholangiocarcinoma. Cell Mol Gastroenterol Hepatol. (2023) 15:985–99. doi: 10.1016/j.jcmgh.2023.01.006, PMID: 36708970 PMC10040968

[5] Yang C Zhang H Zhang L Zhu AX Bernards R Qin W. Evolving therapeutic landscape of advanced hepatocellular carcinoma. Nat Rev Gastroenterol Hepatol. (2023) 20:203–22. doi: 10.1038/s41575-022-00704-9, PMID: 36369487

[6] Chen Y McAndrews KM Kalluri R. Clinical and therapeutic relevance of cancer-associated fibroblasts. Nat Rev Clin Oncol. (2021) 18:792–804. doi: 10.1038/s41571-021-00546-5, PMID: 34489603 PMC8791784

[7] Song M He J Pan Q-Z Yang J Zhao J Zhang Y-J. Cancer-associated fibroblast-mediated cellular crosstalk supports hepatocellular carcinoma progression. Hepatology. (2021) 73:1717–35. doi: 10.1002/hep.31792, PMID: 33682185

[8] Lee JW Stone ML Porrett PM Thomas SK Komar CA Li JH. Hepatocytes direct the formation of a pro-metastatic niche in the liver. Nature. (2019) 567:249–52. doi: 10.1038/s41586-019-1004-y, PMID: 30842658 PMC6430113

[9] Cadamuro M Nuozzi G Simioni P Fabris L. The tumor microenvironment in hepatocarcinoma: dissecting the functions of cancer-associated fibroblasts. hr. (2023) 9:47. doi: 10.20517/2394-5079.2023.94

[10] Eun JW Yoon JH Ahn HR Kim S Kim YB Lim SB. Cancer-associated fibroblast-derived secreted phosphoprotein 1 contributes to resistance of hepatocellular carcinoma to sorafenib and lenvatinib. Cancer Commun (Lond). (2023) 43:455–79. doi: 10.1002/cac2.12414, PMID: 36919193 PMC10091107

[11] Wang X Niu R Yang H Lin Y Hou H Yang H. Fibroblast activation protein promotes progression of hepatocellular carcinoma via regulating the immunity. Cell Biol Int. (2024) 48:577–93. doi: 10.1002/cbin.12154, PMID: 38501437

[12] Murai H Kodama T Maesaka K Tange S Motooka D Suzuki Y. Multiomics identifies the link between intratumor steatosis and the exhausted tumor immune microenvironment in hepatocellular carcinoma. Hepatology. (2023) 77:77–91. doi: 10.1002/hep.32573, PMID: 35567547 PMC9970024

[13] Li Y Hamad M Elkord E. Cancer-associated fibroblasts in hepatocellular carcinoma: heterogeneity, mechanisms and therapeutic targets. Hepatol Int. (2025) 19:325–36. doi: 10.1007/s12072-025-10788-5, PMID: 39979756

[14] Yin Z Dong C Jiang K Xu Z Li R Guo K. Heterogeneity of cancer-associated fibroblasts and roles in the progression, prognosis, and therapy of hepatocellular carcinoma. J Hematol Oncol. (2019) 12:101. doi: 10.1186/s13045-019-0782-x, PMID: 31547836 PMC6757399

[15] Cogliati B Yashaswini CN Wang S Sia D Friedman SL. Friend or foe? The elusive role of hepatic stellate cells in liver cancer. Nat Rev Gastroenterol Hepatol. (2023) 20:647–61. doi: 10.1038/s41575-023-00821-z, PMID: 37550577 PMC10671228

[16] Filliol A Saito Y Nair A Dapito DH Yu L-X Ravichandra A. Opposing roles of hepatic stellate cell subpopulations in hepatocarcinogenesis. Nature. (2022) 610:356–65. doi: 10.1038/s41586-022-05289-6, PMID: 36198802 PMC9949942

[17] Zhou Y Ren H Dai B Li J Shang L Huang J. Hepatocellular carcinoma-derived exosomal miRNA-21 contributes to tumor progression by converting hepatocyte stellate cells to cancer-associated fibroblasts. J Exp Clin Cancer Res. (2018) 37:324. doi: 10.1186/s13046-018-0965-2, PMID: 30591064 PMC6307162

[18] Wang C Shang C Gai X Song T Han S Liu Q. Sulfatase 2-induced cancer-associated fibroblasts promote hepatocellular carcinoma progression via inhibition of apoptosis and induction of epithelial-to-mesenchymal transition. Front Cell Dev Biol. (2021) 9:631931. doi: 10.3389/fcell.2021.631931, PMID: 33889573 PMC8056031

[19] Dranoff JA Wells RG. Portal fibroblasts: Underappreciated mediators of biliary fibrosis. Hepatology. (2010) 51:1438–44. doi: 10.1002/hep.23405, PMID: 20209607 PMC2850946

[20] Xu J Cong M Park TJ Scholten D Brenner DA Kisseleva T. Contribution of bone marrow-derived fibrocytes to liver fibrosis. Hepatobil Surg Nutr. (2015) 4:34–47. doi: 10.3978/j.issn.2304-3881.2015.01.01, PMID: 25713803 PMC4318956

[21] Zeisberg M Yang C Martino M Duncan MB Rieder F Tanjore H. Fibroblasts derive from hepatocytes in liver fibrosis via epithelial to mesenchymal transition. J Biol Chem. (2007) 282:23337–47. doi: 10.1074/jbc.M700194200, PMID: 17562716

[22] Jotzu C Alt E Welte G Li J Hennessy BT Devarajan E. Adipose tissue derived stem cells differentiate into carcinoma-associated fibroblast-like cells under the influence of tumor derived factors. Cell Oncol (Dordr). (2011) 34:55–67. doi: 10.1007/s13402-011-0012-1, PMID: 21327615 PMC13014585

[23] Zeisberg EM Potenta S Xie L Zeisberg M Kalluri R. Discovery of endothelial to mesenchymal transition as a source for carcinoma-associated fibroblasts. Cancer Res. (2007) 67:10123–8. doi: 10.1158/0008-5472.CAN-07-3127, PMID: 17974953

[24] Lua I Li Y Zagory JA Wang KS French SW Sévigny J. Characterization of hepatic stellate cells, portal fibroblasts, and mesothelial cells in normal and fibrotic livers. J Hepatol. (2016) 64:1137–46. doi: 10.1016/j.jhep.2016.01.010, PMID: 26806818 PMC4834254

[25] Baglieri J Brenner DA Kisseleva T. The role of fibrosis and liver-associated fibroblasts in the pathogenesis of hepatocellular carcinoma. Int J Mol Sci. (2019) 20:1723. doi: 10.3390/ijms20071723, PMID: 30959975 PMC6479943

[26] Li Y Wang J Asahina K. Mesothelial cells give rise to hepatic stellate cells and myofibroblasts via mesothelial-mesenchymal transition in liver injury. Proc Natl Acad Sci U.S.A. (2013) 110:2324–9. doi: 10.1073/pnas.1214136110, PMID: 23345421 PMC3568296

[27] Zhang X Li N Zhu Y Wen W. The role of mesenchymal stem cells in the occurrence, development, and therapy of hepatocellular carcinoma. Cancer Med. (2022) 11:931–43. doi: 10.1002/cam4.4521, PMID: 34981659 PMC8855904

[28] Zong C Zhang H Yang X Gao L Hou J Ye F. The distinct roles of mesenchymal stem cells in the initial and progressive stage of hepatocarcinoma. Cell Death Dis. (2018) 9:345. doi: 10.1038/s41419-018-0366-7, PMID: 29497038 PMC5832809

[29] Bhattacharya SD Mi Z Talbot LJ Guo H Kuo PC. Human mesenchymal stem cell and epithelial hepatic carcinoma cell lines in admixture: concurrent stimulation of cancer-associated fibroblasts and epithelial-to-mesenchymal transition markers. Surgery. (2012) 152:449–54. doi: 10.1016/j.surg.2012.06.011, PMID: 22938903 PMC3432987

[30] Pan Y Qiu Y Zhou X Mao W Xu X. Cancer-associated fibroblasts: multidimensional players in liver cancer. Front Oncol. (2025) 15:1454546. doi: 10.3389/fonc.2025.1454546, PMID: 40248197 PMC12003132

[31] Wang H Liu F Wu X Zhu G Tang Z Qu W. Cancer-associated fibroblasts contributed to hepatocellular carcinoma recurrence and metastasis via CD36-mediated fatty-acid metabolic reprogramming. Exp Cell Res. (2024) 435:113947. doi: 10.1016/j.yexcr.2024.113947, PMID: 38301989

[32] Long F Zhong W Zhao F Xu Y Hu X Jia G. DAB2 + macrophages support FAP + fibroblasts in shaping tumor barrier and inducing poor clinical outcomes in liver cancer. Theranostics. (2024) 14:4822–43. doi: 10.7150/thno.99046, PMID: 39239526 PMC11373629

[33] Sun L Wang Y Wang L Yao B Chen T Li Q. Resolvin D1 prevents epithelial-mesenchymal transition and reduces the stemness features of hepatocellular carcinoma by inhibiting paracrine of cancer-associated fibroblast-derived COMP. J Exp Clin Cancer Res. (2019) 38:170. doi: 10.1186/s13046-019-1163-6, PMID: 30999932 PMC6472102

[34] Liu C Liu L Chen X Cheng J Zhang H Zhang C. LSD1 stimulates cancer-associated fibroblasts to drive notch3-dependent self-renewal of liver cancer stem-like cells. Cancer Res. (2018) 78:938–49. doi: 10.1158/0008-5472.CAN-17-1236, PMID: 29259010

[35] Meng Y Sang Y Liao J Zhao Q Qu S Li R. Single cell transcriptional diversity and intercellular crosstalk of human liver cancer. Cell Death Dis. (2022) 13:261. doi: 10.1038/s41419-022-04689-w, PMID: 35322024 PMC8943132

[36] Zhu G-Q Tang Z Huang R Qu W-F Fang Y Yang R. CD36+ cancer-associated fibroblasts provide immunosuppressive microenvironment for hepatocellular carcinoma via secretion of macrophage migration inhibitory factor. Cell Discov. (2023) 9:25. doi: 10.1038/s41421-023-00529-z, PMID: 36878933 PMC9988869

[37] Wang H Liang Y Liu Z Zhang R Chao J Wang M. POSTN+ cancer-associated fibroblasts determine the efficacy of immunotherapy in hepatocellular carcinoma. J Immunother Cancer. (2024) 12:e008721. doi: 10.1136/jitc-2023-008721, PMID: 39067872 PMC11284881

[38] Liu Y Dong G Yu J Liang P. Integration of single-cell and spatial transcriptomics reveals fibroblast subtypes in hepatocellular carcinoma: spatial distribution, differentiation trajectories, and therapeutic potential. J Transl Med. (2025) 23:198. doi: 10.1186/s12967-025-06192-0, PMID: 39966876 PMC11837652

[39] Chiavarina B Ronca R Otaka Y Sutton RB Rezzola S Yokobori T. Fibroblast-derived prolargin is a tumor suppressor in hepatocellular carcinoma. Oncogene. (2022) 41:1410–20. doi: 10.1038/s41388-021-02171-z, PMID: 35031773

[40] Yan W Xiao G-H Wang L-J Zhou Y Yang F Mou K-H. CAFs activated by YAP1 upregulate cancer matrix stiffness to mediate hepatocellular carcinoma progression. J Transl Med. (2025) 23:450. doi: 10.1186/s12967-025-06325-5, PMID: 40241143 PMC12004581

[41] Liu Z Chen M Zhao R Huang Y Liu F Li B. CAF-induced placental growth factor facilitates neoangiogenesis in hepatocellular carcinoma. Acta Biochim Biophys Sin (Shanghai). (2020) 52:18–25. doi: 10.1093/abbs/gmz134, PMID: 31828297

[42] She Q Hu S Pu X Guo Q Mou C Yang C. The effect of hepatocellular carcinoma-associated fibroblasts on hepatoma vasculogenic mimicry. Am J Cancer Res. (2020) 10:4198–210., PMID: 33414995 PMC7783763

[43] Liu G Yang Z-F Sun J Sun B-Y Zhou P-Y Zhou C. The LINC00152/miR-205-5p/CXCL11 axis in hepatocellular carcinoma cancer-associated fibroblasts affects cancer cell phenotypes and tumor growth. Cell Oncol (Dordr). (2022) 45:1435–49. doi: 10.1007/s13402-022-00730-4, PMID: 36435866 PMC9747837

[44] Zhang Z Li X Sun W Yue S Yang J Li J. Loss of exosomal miR-320a from cancer-associated fibroblasts contributes to HCC proliferation and metastasis. Cancer Lett. (2017) 397:33–42. doi: 10.1016/j.canlet.2017.03.004, PMID: 28288874

[45] Shang H Lu L Fan M Lu Y Shi X Lu H. Exosomal circHIF1A derived from hypoxic-induced carcinoma-associated fibroblasts promotes hepatocellular carcinoma cell Malignant phenotypes and immune escape. Int Immunopharmacol. (2024) 138:112282. doi: 10.1016/j.intimp.2024.112282, PMID: 38936058

[46] Luo Q Wang C-Q Yang L-Y Gao X-M Sun H-T Zhang Y. FOXQ1/NDRG1 axis exacerbates hepatocellular carcinoma initiation via enhancing crosstalk between fibroblasts and tumor cells. Cancer Lett. (2018) 417:21–34. doi: 10.1016/j.canlet.2017.12.021, PMID: 29248714

[47] Zulaziz N Chai SJ Lim KP. The origins, roles and therapies of cancer associated fibroblast in liver cancer. Front Oncol. (2023) 13:1151373. doi: 10.3389/fonc.2023.1151373, PMID: 37035187 PMC10076538

[48] Yang J Lu Y Lin Y-Y Zheng Z-Y Fang J-H He S. Vascular mimicry formation is promoted by paracrine TGF-β and SDF1 of cancer-associated fibroblasts and inhibited by miR-101 in hepatocellular carcinoma. Cancer Lett. (2016) 383:18–27. doi: 10.1016/j.canlet.2016.09.012, PMID: 27693460

[49] Schrader J Gordon-Walker TT Aucott RL van Deemter M Quaas A Walsh S. Matrix stiffness modulates proliferation, chemotherapeutic response, and dormancy in hepatocellular carcinoma cells. Hepatology. (2011) 53:1192–205. doi: 10.1002/hep.24108, PMID: 21442631 PMC3076070

[50] Peng Z Ding Y Zhang H Meng X Huang Y Zhang P. Mechanical force-mediated interactions between cancer cells and fibroblasts and their role in the progression of hepatocellular carcinoma. jcmt. (2024) 10:N/A–A. doi: 10.20517/2394-4722.2023.137

[51] Jung W-H Yam N Chen C-C Elawad K Hu B Chen Y. Force-dependent extracellular matrix remodeling by early-stage cancer cells alters diffusion and induces carcinoma-associated fibroblasts. Biomaterials. (2020) 234:119756. doi: 10.1016/j.biomaterials.2020.119756, PMID: 31954229

[52] Peng H Yang M Feng K Lv Q Zhang Y. Semaphorin 3C (Sema3C) reshapes stromal microenvironment to promote hepatocellular carcinoma progression. Signal Transduct Target Ther. (2024) 9:169. doi: 10.1038/s41392-024-01887-0, PMID: 38956074 PMC11220018

[53] Xu H Zhao J Li J Zhu Z Cui Z Liu R. Cancer associated fibroblast-derived CCL5 promotes hepatocellular carcinoma metastasis through activating HIF1α/ZEB1 axis. Cell Death Dis. (2022) 13:478. doi: 10.1038/s41419-022-04935-1, PMID: 35589690 PMC9119971

[54] Fang T Lv H Lv G Li T Wang C Han Q. Tumor-derived exosomal miR-1247-3p induces cancer-associated fibroblast activation to foster lung metastasis of liver cancer. Nat Commun. (2018) 9:191. doi: 10.1038/s41467-017-02583-0, PMID: 29335551 PMC5768693

[55] Liu G Sun J Yang Z-F Zhou C Zhou P-Y Guan R-Y. Cancer-associated fibroblast-derived CXCL11 modulates hepatocellular carcinoma cell migration and tumor metastasis through the circUBAP2/miR-4756/IFIT1/3 axis. Cell Death Dis. (2021) 12:260. doi: 10.1038/s41419-021-03545-7, PMID: 33707417 PMC7952559

[56] Li Y Wang R Xiong S Wang X Zhao Z Bai S. Cancer-associated fibroblasts promote the stemness of CD24+ liver cells via paracrine signaling. J Mol Med (Berl). (2019) 97:243–55. doi: 10.1007/s00109-018-1731-9, PMID: 30564864

[57] Xiong S Wang R Chen Q Luo J Wang J Zhao Z. Cancer-associated fibroblasts promote stem cell-like properties of hepatocellular carcinoma cells through IL-6/STAT3/Notch signaling. Am J Cancer Res. (2018) 8:302–16., PMID: 29511600 PMC5835697

[58] Peng H Xue R Ju Z Qiu J Wang J Yan W. Cancer-associated fibroblasts enhance the chemoresistance of CD73+ hepatocellular carcinoma cancer cells via HGF-Met-ERK1/2 pathway. Ann Transl Med. (2020) 8:856. doi: 10.21037/atm-20-1038, PMID: 32793700 PMC7396767

[59] Lau EYT Lo J Cheng BYL Ma MKF Lee JMF Ng JKY. Cancer-Associated Fibroblasts Regulate Tumor-Initiating Cell Plasticity in Hepatocellular Carcinoma through c-Met/FRA1/HEY1 Signaling. Cell Rep. (2016) 15:1175–89. doi: 10.1016/j.celrep.2016.04.019, PMID: 27134167

[60] Loh J-J Li T-W Zhou L Wong T-L Liu X Ma VWS. FSTL1 secreted by activated fibroblasts promotes hepatocellular carcinoma metastasis and stemness. Cancer Res. (2021) 81:5692–705. doi: 10.1158/0008-5472.CAN-20-4226, PMID: 34551961

[61] Jing S-Y Liu D Feng N Dong H Wang H-Q Yan X. Spatial multiomics reveals a subpopulation of fibroblasts associated with cancer stemness in human hepatocellular carcinoma. Genome Med. (2024) 16:98. doi: 10.1186/s13073-024-01367-8, PMID: 39138551 PMC11320883

[62] Su Z Lu C Zhang F Liu H Li M Qiao M. Cancer-associated fibroblasts-secreted exosomal miR-92a-3p promotes tumor growth and stemness in hepatocellular carcinoma through activation of Wnt/β-catenin signaling pathway by suppressing AXIN1. J Cell Physiol. (2024) 239:e31344. doi: 10.1002/jcp.31344, PMID: 38949237

[63] Cheng J-T Deng Y-N Yi H-M Wang G-Y Fu B-S Chen W-J. Hepatic carcinoma-associated fibroblasts induce IDO-producing regulatory dendritic cells through IL-6-mediated STAT3 activation. Oncogenesis. (2016) 5:e198. doi: 10.1038/oncsis.2016.7, PMID: 26900950 PMC5154347

[64] Deng Y Cheng J Fu B Liu W Chen G Zhang Q. Hepatic carcinoma-associated fibroblasts enhance immune suppression by facilitating the generation of myeloid-derived suppressor cells. Oncogene. (2017) 36:1090–101. doi: 10.1038/onc.2016.273, PMID: 27593937

[65] Cheng Y Li H Deng Y Tai Y Zeng K Zhang Y. Cancer-associated fibroblasts induce PDL1+ neutrophils through the IL6-STAT3 pathway that foster immune suppression in hepatocellular carcinoma. Cell Death Dis. (2018) 9:422. doi: 10.1038/s41419-018-0458-4, PMID: 29556041 PMC5859264

[66] Lin Y Li B Yang X Cai Q Liu W Tian M. Fibroblastic FAP promotes intrahepatic cholangiocarcinoma growth via MDSCs recruitment. Neoplasia. (2019) 21:1133–42. doi: 10.1016/j.neo.2019.10.005, PMID: 31759251 PMC6880109

[67] Yang X Lin Y Shi Y Li B Liu W Yin W. FAP promotes immunosuppression by cancer-associated fibroblasts in the tumor microenvironment via STAT3-CCL2 signaling. Cancer Res. (2016) 76:4124–35. doi: 10.1158/0008-5472.CAN-15-2973, PMID: 27216177

[68] Chen S Morine Y Tokuda K Yamada S Saito Y Nishi M. Cancer−associated fibroblast−induced M2−polarized macrophages promote hepatocellular carcinoma progression via the plasminogen activator inhibitor−1 pathway. Int J Oncol. (2021) 59:59. doi: 10.3892/ijo.2021.5239, PMID: 34195849 PMC8253588

[69] Mun K Han J Roh P Park J Kim G Hur W. Isolation and characterization of cancer-associated fibroblasts in the tumor microenvironment of hepatocellular carcinoma. J Liver Cancer. (2023) 23:341–9. doi: 10.17998/jlc.2023.04.30, PMID: 37488925 PMC10565539

[70] Yang F Wei Y Han D Li Y Shi S Jiao D. Interaction with CD68 and regulation of GAS6 expression by endosialin in fibroblasts drives recruitment and polarization of macrophages in hepatocellular carcinoma. Cancer Res. (2020) 80:3892–905. doi: 10.1158/0008-5472.CAN-19-2691, PMID: 32591411

[71] Liu H Shen J Lu K. IL-6 and PD-L1 blockade combination inhibits hepatocellular carcinoma cancer development in mouse model. Biochem Biophys Res Commun. (2017) 486:239–44. doi: 10.1016/j.bbrc.2017.02.128, PMID: 28254435

[72] Mazzocca A Fransvea E Dituri F Lupo L Antonaci S Giannelli G. Down-regulation of connective tissue growth factor by inhibition of transforming growth factor beta blocks the tumor-stroma cross-talk and tumor progression in hepatocellular carcinoma. Hepatology. (2010) 51:523–34. doi: 10.1002/hep.23285, PMID: 19821534

[73] Cheng A-L Thongprasert S Lim HY Sukeepaisarnjaroen W Yang T-S Wu C-C. Randomized, open-label phase 2 study comparing frontline dovitinib versus sorafenib in patients with advanced hepatocellular carcinoma. Hepatology. (2016) 64:774–84. doi: 10.1002/hep.28600, PMID: 27082062

[74] Macarulla T Moreno V Chen L-T Sawyer MB Goyal L Muñoz Martín AJ. Phase I study of H3B-6527 in hepatocellular carcinoma (HCC) or intrahepatic cholangiocarcinoma (ICC). JCO. (2021) 39:4090–0. doi: 10.1200/JCO.2021.39.15_suppl.4090

[75] Kelley RK Gane E Assenat E Siebler J Galle PR Merle P. A phase 2 study of galunisertib (TGF-β1 receptor type I inhibitor) and sorafenib in patients with advanced hepatocellular carcinoma. Clin Transl Gastroenterol. (2019) 10:e00056. doi: 10.14309/ctg.0000000000000056, PMID: 31295152 PMC6708671

[76] Yi M Niu M Wu Y Ge H Jiao D Zhu S. Combination of oral STING agonist MSA-2 and anti-TGF-β/PD-L1 bispecific antibody YM101: a novel immune cocktail therapy for non-inflamed tumors. J Hematol Oncol. (2022) 15:142. doi: 10.1186/s13045-022-01363-8, PMID: 36209176 PMC9548169

[77] Zhou M Zhu S Xu C Liu B Shen J. A phase Ib/II study of BLU-554, a fibroblast growth factor receptor 4 inhibitor in combination with CS1001, an anti-PD-L1, in patients with locally advanced or metastatic hepatocellular carcinoma. Invest New Drugs. (2023) 41:162–7. doi: 10.1007/s10637-023-01335-w, PMID: 36763233

